# Birth Asphyxia Is Associated With Increased Risk of Cerebral Palsy: A Meta-Analysis

**DOI:** 10.3389/fneur.2020.00704

**Published:** 2020-07-16

**Authors:** Shan Zhang, Bingbing Li, Xiaoli Zhang, Changlian Zhu, Xiaoyang Wang

**Affiliations:** ^1^Henan Key Laboratory of Child Brain Injury, Third Affiliated Hospital and Institute of Neuroscience of Zhengzhou University, Zhengzhou, China; ^2^Center for Brain Repair and Rehabilitation, Institute of Neuroscience and Physiology, Sahlgrenska Academy, University of Gothenburg, Gothenburg, Sweden; ^3^Department of Women's and Children's Health, Karolinska Institutet, Stockholm, Sweden; ^4^Center of Perinatal Medicine and Health, Institute of Neuroscience and Physiology, Sahlgrenska Academy, University of Gothenburg, Gothenburg, Sweden

**Keywords:** birth asphyxia, cerebral palsy, erythropoietin, hypothermia, meta-analysis

## Abstract

**Objective:** To assess the association between birth asphyxia—as defined by the pH of umbilical cord blood—and cerebral palsy in asphyxiated neonates ≥35 weeks' gestation.

**Methods:** Two reviewers independently selected English-language studies that included data on the incidence of cerebral palsy in asphyxiated neonates ≥35 weeks' gestation. Studies were searched from the Embase, Google Scholar, PubMed, and Cochrane Library databases up to 31 December 2019, and the references in the retrieved articles were screened.

**Results:** We identified 10 studies that met the inclusion criteria for our meta-analysis, including 8 randomized controlled trials and 2 observational studies. According to a random effects model, the pooled rate of cerebral palsy in the randomized controlled trials was 20.3% (95% CI: 16.0–24.5) and the incidence of cerebral palsy in the observational studies was 22.2% (95% CI: 8.5–35.8). Subgroup analysis by treatment for hypoxic ischemic encephalopathy in asphyxiated neonates showed that the pooled rates of cerebral palsy were 17.3% (95% CI: 13.3–21.2) and 23.9% (95% CI: 18.1–29.7) for the intervention group and non-intervention group, respectively.

**Conclusion:** Our findings suggest that the incidence of cerebral palsy in neonates (≥35 weeks' gestation) with perinatal asphyxia is significantly higher compared to that in the healthy neonate population. With the growing emphasis on improving neonatal neurodevelopment and reducing neurological sequelae, we conclude that the prevention and treatment of perinatal asphyxia is essential for preventing the development of cerebral palsy.

## Introduction

Cerebral palsy (CP) is a group of syndromes caused by non-progressive brain injury in the fetus or infant and leads to lifelong disability (1). The prevalence of CP is 2.11 per 1,000 live births globally (2), which remained relatively stable from 1950 to 1980, but increased moderately between 1980 and 1990, probably due to the increased survival of very premature infants as a result of improvements in perinatal care (3, 4). The etiology of CP is complex and multifactorial (5). Although premature birth is a risk factor, the causes of CP for children born at term remain unclear (6–8). Birth asphyxia has been involved, but its contribution to CP is debatable. Evidence has suggested that most cases of CP are caused by prenatal factors and that the role of birth asphyxia is relatively small (<10% of cases) (9, 10). However, other studies have shown that birth asphyxia is one of the main causes of CP, accounting for more than 30% of cases (11–13).

Birth asphyxia is one of the important causes of neonatal morbidity and mortality (14, 15). Birth asphyxia refers to interruption of the blood flow to the placenta, leading to hypoxia and ischemia. When hypoxia–ischemia persists long enough, it will cause permanent neurologic injury, which may eventually develop into neurodevelopmental disorders such as developmental delay and CP (16, 17). The inconsistencies in the diagnosis of birth asphyxia contribute to variation in the prognosis of birth asphyxia. A previous study found that in studies with different diagnostic criteria of birth asphyxia, the proportion of CP cases with birth asphyxia ranged from <3% to over 50% (18). Metabolic acidosis in the umbilical cord has been recognized internationally as a necessary criterion for defining intrapartum hypoxia (19, 20) and has been used as the definition of asphyxia (21). Thus, the pH value of umbilical cord blood was used as the diagnostic criteria of birth asphyxia for conducting a meta-analysis of human studies to investigate the case exposure rates linking birth asphyxia to CP.

## Methods

### Literature Search

Relevant studies were searched from the PubMed, Google Scholar, Embase, and Cochrane Library databases up to 31 December 2019. The search was performed using keywords and subject terms related to “birth asphyxia.” The keywords and subject terms related to “cerebral palsy” or “neurodevelopmental outcome” were used to acquire studies related to CP. We combined the two parts of the search terms using “AND” to retrieve the studies (Supplementary Table 1). In order to supplement the electronic searches, we also searched the reference lists of previous reviews, key papers, and other relevant literature screened by the electronic search. Two investigators independently reviewed the titles, abstracts, and full-text publications.

### Inclusion Criteria

Eligible studies were limited to research focusing on the following: (1) newborn infants who were born at ≥35 weeks' gestation, (2) evidence of birth asphyxia based on a pH ≤ 7.0 and/or a base deficit ≥12 mmol/L in an umbilical cord blood sample during the first hour after birth, and (3) clinical hypoxic ischemic encephalopathy (HIE) manifestation as well as neurodevelopmental outcomes that included data on CP. Additionally, when multiple studies based on the same population were published, only the most complete one was included.

### Exclusion Criteria

The following exclusion criteria were applied: (1) reviews, meta-analyses, or case reports, (2) studies not published in English, (3) studies using evidence for birth asphyxia that was inconsistent with our inclusion criteria described above, and (4) studies reporting overlapping data.

### Data Extraction

Information relating to data extraction was gathered individually from each identified article, including the name of the first author, study design, publication year, the size of sample, gestational age, asphyxiation criteria, follow-up period, and neurodevelopmental outcome regarding CP.

### Quality Assessment

Of the included studies, eight were randomized controlled trials and two were observational studies. The Newcastle-Ottawa Scale was used to evaluate the quality of the observational studies (22) (Supplementary Table 2), including the choice of the research population (0–4 points), the study comparability (0–2 points), and the evaluation of exposure factor and outcome (0–3 points). The Cochrane collaboration's tool for assessing risk of bias, which is based on the important elements of reducing bias, including selection bias, performance bias, detection bias, attrition bias, reporting bias, and other biases, was used to assess the quality of the randomized controlled trials (23) (Supplementary Figure 1).

### Statistical Analysis

Stata software version 12.0 (Stata Corporation, College Station, TX, US) was used for data analysis. The combined rate of CP and 95% confidence intervals (CIs) were calculated for all predetermined groups. A random effects model was used to give a pooled estimate of prevalence because of the small number of studies and the heterogeneity across studies in this meta-analysis. Heterogeneity was estimated by the Q statistic and the *I*^2^ statistic. Sensitivity analyses were performed to identify any potential influence between the included studies on the pooled prevalence of CP. Possible publication bias was tested by Egger's and Begg's tests. The significance level of Q statistic for the heterogeneity test was set to 0.10 and *p* < 0.05 were considered statistically significant.

## Results

### Search Results

**Figure 1:**
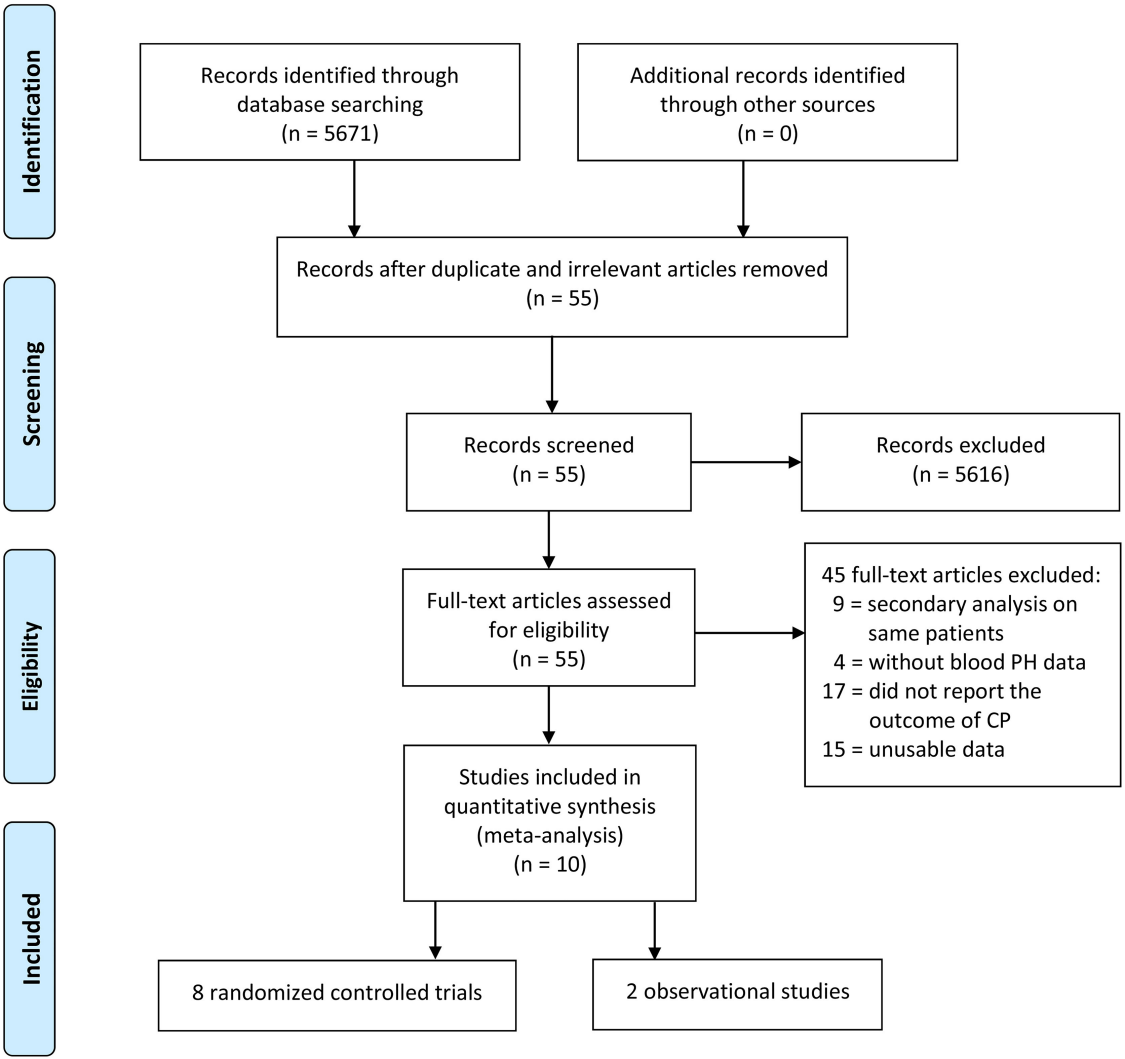
Flow chart for study selection.

The electronic database searches initially yielded 5,671 studies, and 5,616 studies were deleted due to either repetition or lack of relevance. A total of 55 full-text studies were retrieved and critically appraised. Of these articles, 45 did not satisfy the inclusion criteria (9 studies performed secondary analyses on the same study populations, 17 studies did not report the neurodevelopment outcomes, 4 studies did not have data for the pH of umbilical cord blood, and 15 studies were excluded due to unusable data such as articles that reported the prevalence of CP between those with birth asphyxia and those without birth asphyxia and articles for which the exact association between asphyxia and CP could not be determined). Of the remaining 10 acceptable studies, 8 studies were randomized controlled trials and 2 studies were observational studies (Figure 1).

### Characteristics of the Studies

We compiled a dataset of 1,665 infants from the 10 studies. All of the neonates in the included studies meeting the asphyxia criteria were diagnosed as HIE. Of the eight randomized controlled trials (24–31), six studies used moderate hypothermia as a treatment for HIE in asphyxiated neonates, one used erythropoietin treatment in asphyxiated neonates, and one used a combination of moderate hypothermia and topiramate treatment in asphyxiated neonates. The two observational studies (32, 33) investigated the neurodevelopment outcomes in asphyxiated neonates without any interventions. The characteristics of included trials were summarized in Table 1.

**Table 1:** Characteristics of the included studies.

**Study**	**Publication year**	**Study design**	***N***	**Gestational age, weeks**	**Umbilical cord blood pH value**	**Number of CP cases**	**Follow-up period**
Carli et al. (32)	2004	Cohort study	43	≥37	pH < 7.00 or BD ≥ 12 mmol/L	13	12–36 months
Gluckman et al. (24)	2005	RCT	218	≥36	pH < 7.00 or BD > 16 mmol/L	52	18 months
Shankaran et al. (25)	2005	RCT	205	≥36	pH ≤ 7.00 or BD ≥ 16 mmol/L	34	18–22 months
Azzopardi et al. (26)	2009	RCT	323	≥36	pH < 7.00 or BD ≥ 16 mmol/L	81	18 months
Simbruner et al. (27)	2010	RCT	111	≥36	pH < 7.00 or BD > 16 mmol/L	14	18–21 months
Jacobs et al. (28)	2011	RCT	208	≥35	pH < 7.00 or BD ≥ 12 mmol/L	38	24 months
Perez et al. (33)	2013	Cohort study	68	≥36	pH < 7.1 and BD < −10 mmol/L	11	8.2–15.7 years
Filippi et al. (29)	2017	RCT	42	≥36	pH < 7.00 and/or BD > 16 mmol/L	13	18–24 months
Malla et al. (30)	2017	RCT	100	≥37	pH ≤ 7.00 and/or BD ≥ 16 mmol/L	29	19 months
Shankaran et al. (31)	2017	RCT	347	≥36	pH ≤ 7.00 or BD ≥ 16 mmol/L	47	18–22 months

*BD, base deficit*.

### Sensitivity Analysis and Publication Bias

Sensitivity analysis was conducted on the eight randomized controlled trials, and none of them had a significant impact on the results of the meta-analysis, suggesting that this study had good stability (Supplementary Figure 2). Publication bias was evaluated by Egger's (*P* = 0.134) and Begg's (*P* = 0.536) tests (Supplementary Figure 3). The pooled results demonstrated that there was no significant publication bias.

### Pooled Rate of CP

**Figure 2:**
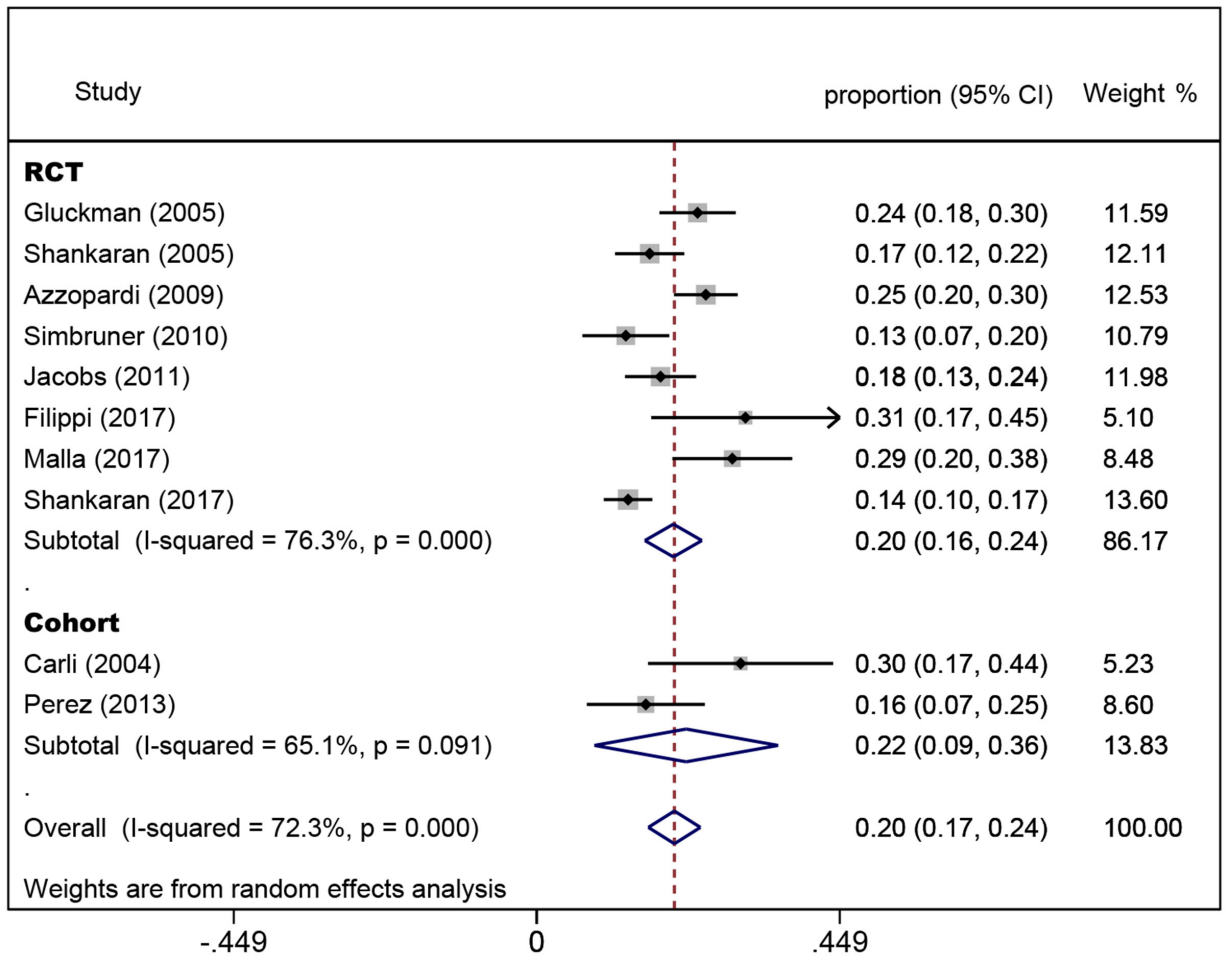
Forest plot of the pooled rate of cerebral palsy. The solid diamonds and horizontal solid lines represent the proportions and 95% CIs of each included study. The size of the gray area indicates the study-specific statistical weight. The hollow diamonds show the pooled proportions and 95% CIs of each group and the overall population. The vertical red dotted line shows the combined effect estimate.

**Figure 3:**
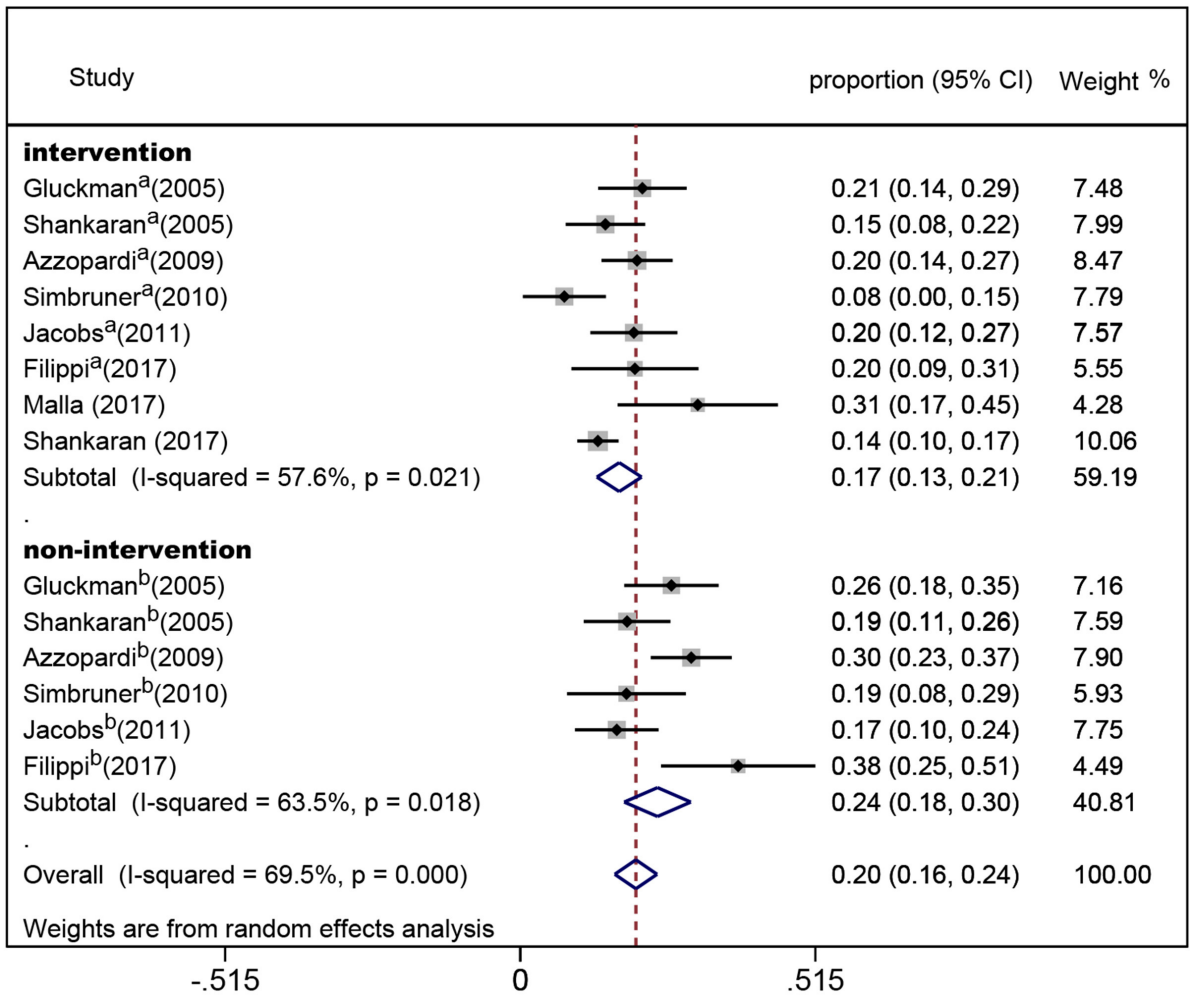
Forest plot of the pooled rate of cerebral palsy in the intervention and non-intervention groups in randomized controlled trials. The solid diamonds and horizontal solid lines represent the proportions and 95% CIs of each included study. The size of the gray area indicates the study-specific statistical weight. The hollow diamonds show the pooled proportions and 95% CIs of each subgroup and the overall population. The vertical red dotted line shows the combined effect estimate.

In the eight randomized controlled trials, the number of infants with CP was 308 for a pooled rate of 20.3% (95% CI: 16.0–24.5, *I*^2^ = 76.3%). In the two observational studies, the number of infants with CP was 24 and the combined incidence was 22.2% (95% CI: 8.5–35.8, *I*^2^ = 65.1%) (Figure 2). In the randomized controlled trials, the infants were divided into intervention and non-intervention groups. The number of infants with CP was 166 in the intervention group and was 142 in the non-intervention group. The pooled rate of CP was 17.3% (95% CI: 13.3–21.2, *I*^2^ = 57.6%) in the intervention group and was 23.9% (95% CI: 18.1–29.7, *I*^2^ = 63.5%) in the non-intervention group (Figure 3), indicating that whether interventions were performed or not led to the high heterogeneity between studies.

## Discussion

To our knowledge, this is the first evaluation of the link between birth asphyxia and CP using the pH value of umbilical cord blood as a diagnostic criterion for birth asphyxia in addition to clinical HIE manifestations. The results of this meta-analysis indicated that birth asphyxia is associated with CP in both term and near-term infants.

Birth asphyxia might affect the outcomes of neurodevelopment in infants through a variety of mechanisms. Prolonged or intense asphyxia will cause energy depletion in tissues that depend on aerobic metabolism, such as the central nervous system (34, 35). Lack of energy can lead to the failure of ATP-dependent pumps resulting in the loss of neuronal transmembrane potential (36), and thus the most sensitive areas of the brain will die (37–39). In areas that are more resistant, excessive excitability of neurons, abundant ionic calcium influx, free radical generation, and changes in mitochondrial metabolism (40–42) might cause secondary energy exhaustion and programmed neuronal death (43, 44). Thus, these irreversible brain injuries during early brain development might ultimately result in CP.

The accurate diagnosis of birth asphyxia is still a challenge worldwide, resulting in an unclear correlation between asphyxia and CP. Birth asphyxia is predicated by fetal metabolic acidosis, as measured by umbilical cord pH at birth (21, 45), and a recent study showed that a low umbilical cord pH was associated with the occurrence of CP but failed to prove that there was a link between the degree of acidosis and the prevalence or severity of CP (46). In two studies an umbilical arterial pH ≤ 7.00 was referred to pathological or severe fetal acidemia in which the risk of adverse neurological sequelae was increased (47, 48). Furthermore, different consensus statements have mentioned the diagnosis of intrapartum asphyxia since 1992. These statements point out that metabolic acidosis (pH < 7.0 and base deficit of 12 mmol/L or more) is the essential criterion for the diagnosis of asphyxia (49, 50). Therefore, an umbilical arterial pH ≤ 7.00 and/or a base deficit of 12 mmol/L or more was used as the standard of acidosis to diagnose birth asphyxia in this meta-analysis in addition to neonatal clinical HIE manifestations.

The American Academy of Pediatrics and the Society of Obstetrics and Gynecology suggests that infants suffering from “asphyxiation” near delivery, which is severe enough to result in acute neurologic injury, should meet the following criteria: (1) severe metabolic or mixed acidemia (pH < 7.00) on an umbilical arterial blood sample, (2) an Apgar score of 0 to 3 for longer than 5 min, (3) neurologic manifestation such as seizure, coma, or hypotonia, and (4) evidence of multiorgan dysfunction (51). However, it is difficult to measure all of the diagnostic criteria in the clinic, and currently the Apgar score system is most commonly used. Neither Apgar score nor umbilical arterial pH ≤ 7.00 and/or a base deficit ≥ 12 mmol/L as the diagnostic criterion of asphyxia is a complete definition of birth asphyxia, and fetal severe acidosis is instead considered to be a fairer and more objective standard (52).

The evidence reported in previous studies was unable to support a clear association between birth asphyxia and CP (9, 53). However, our pooled analysis of 1,665 infants in 10 studies showed that the CP incidence was 20.3% (95% CI: 16.0–24.5, *I*^2^ = 76.3%) in the randomized controlled trials and 22.2% (95% CI: 8.5–35.8, *I*^2^ = 65.1%) in the observational studies, which means a more relevant association between birth asphyxia and CP. With the popularization of blood gas analysis, the detection of umbilical arterial pH value at birth is easier to perform (54). Our results suggest that blood gas analysis should be used along with Apgar score in daily clinic work when there is the possibility of birth asphyxia. Considering that preterm birth is a risk factor of CP (6, 7), we only included studies with newborn infants who were born at term or near term. Some of the patients were treated with hypothermia and/or drugs in randomized controlled trials, so we divided these patients into intervention and non-intervention groups. The results showed that the incidence of CP in the intervention group had a slight decrease compared to that in the non-intervention group, which was consistent with the conclusion that hypothermia therapy can reduce the risk of neurological impairment in infants with HIE (55). Further treatment should be considered to prevent CP after hypothermia in the acute phase (56).

Previous analysis the association of birth asphyxia and CP showed a large variation from 3 to 50% (18). Other researchers also indicated that <12% of children who have CP were exposed to perinatal asphyxia, which contradicts our results (57–59). For such inconsistent results linking birth asphyxia and CP, the differences in the diagnosis criteria of birth asphyxia were probably the main problems (5, 60). The critical criteria might affect our results to a certain extent. Nevertheless, we still hope to emphasize the importance of severe acidosis as one of necessary criteria of asphyxia in clinical application through the results in this meta-analysis.

Our meta-analysis has some other limitations. First, we only searched literature published in English. Second, publication bias and incomplete ascertainment of published literature might exist. Third, the number of studies in our analysis was small, and the selection of the diagnostic criteria of birth asphyxia might have caused a selection bias in our study. Therefore, the results of this study should be interpreted with caution. Furthermore, some of the included studies used interventions, so measurement bias existed and some heterogeneity was inevitable.

In conclusion, our meta-analysis provides evidence that birth asphyxia is associated with CP in children. Thus, the prevention and treatment of birth asphyxia is of great significance for reducing the prevalence of CP.

## Data Availability Statement

All datasets presented in this study are included in the article/Supplementary Material.

## Author Contributions

SZ and BL searched the databases, screened the articles, and collected the data. SZ wrote the first draft of the manuscript. BL and XZ were responsible for the statistical analysis and interpretation of the data. XW coordinated and supervised the data collection. SZ, CZ, and XW participated in study conception and design. XZ, CZ, and XW critically reviewed and revised the manuscript. All authors contributed to and approved the final version. All authors contributed to the article and approved the submitted version.

## Conflict of Interest

The authors declare that the research was conducted in the absence of any commercial or financial relationships that could be construed as a potential conflict of interest.
