# Supplementary material for: Birth Asphyxia Is Associated With Increased Risk of Cerebral Palsy: A Meta-Analysis
Source: Front Neurol. 2020 Jul 16;11:704. doi: 10.3389/fneur.2020.00704 (PMC7381116; doi:10.3389/fneur.2020.00704)
Supplement: Supplementary file 1 [file Data_Sheet_1.docx]

**Suppl. Table S1.** Search terms used in the literature search

| Birth asphyxia | Cerebral palsy | Neurodevelopmental outcome |
| --- | --- | --- |
| Neonatal asphyxia | CP | Long-term outcome |
| Perinatal asphyxia | Little Disease | Neurological outcome |
| Intrapartum asphyxia | Cerebral palsy | Neurodevelopment |
| Asphyxia Neonatorum | Quadripleg* | Neurological impairment |
| Hypoxia, brain or umbilical  cord blood | Spastic* |  |
| Birth asphyxia | Cerebral pals* |  |

**Suppl. Table S2.** Newcastle-Ottawa Scale quality assessment for the observational studies

| Studies | Quality assessment criteria | | | |
| --- | --- | --- | --- | --- |
|  | Selection | Comparability | Outcome | Overall quality |
| Carli 2004 | ★★★ | ★ | ★★ | 6 |
| Perez 2013 | ★★★ | ★★ | ★★ | 7 |
