# Supplementary figures and images for: Birth Asphyxia Is Associated With Increased Risk of Cerebral Palsy: A Meta-Analysis
Source: Front Neurol. 2020 Jul 16;11:704. doi: 10.3389/fneur.2020.00704 (PMC7381116; doi:10.3389/fneur.2020.00704)

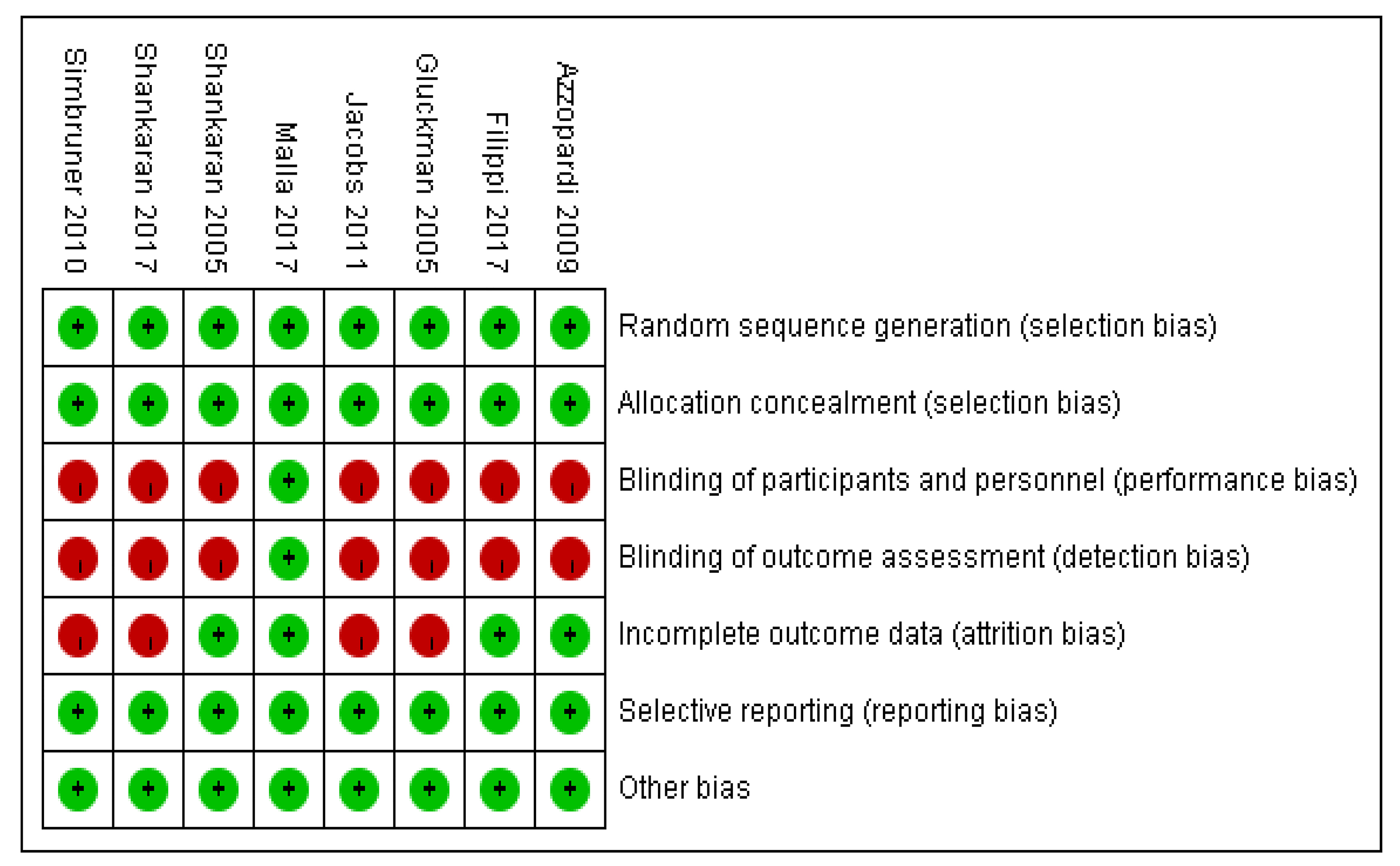

Supplement: Supplementary file 2 [file Image_1.JPEG]

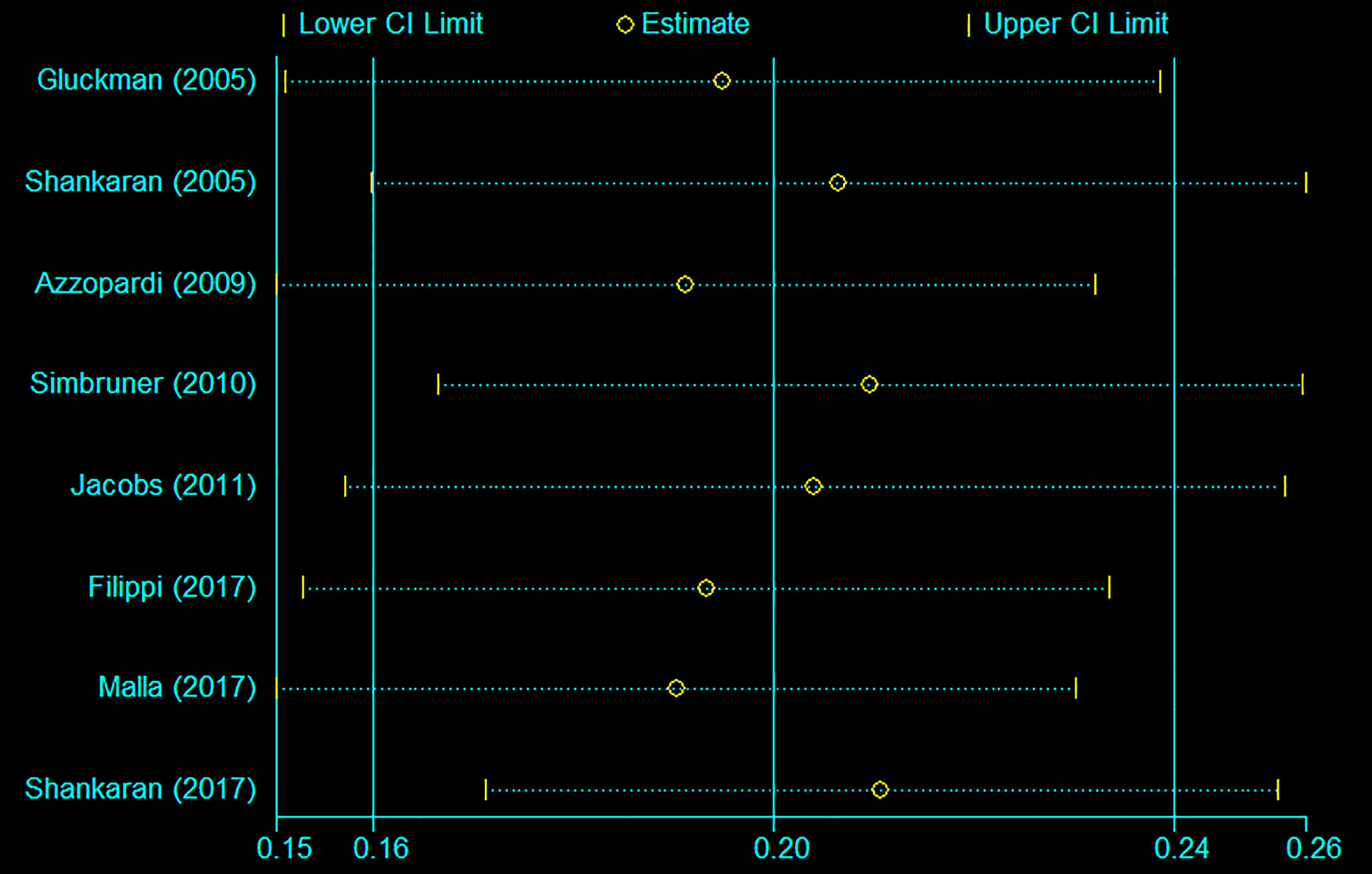

Supplement: Supplementary file 3 [file Image_2.TIF]

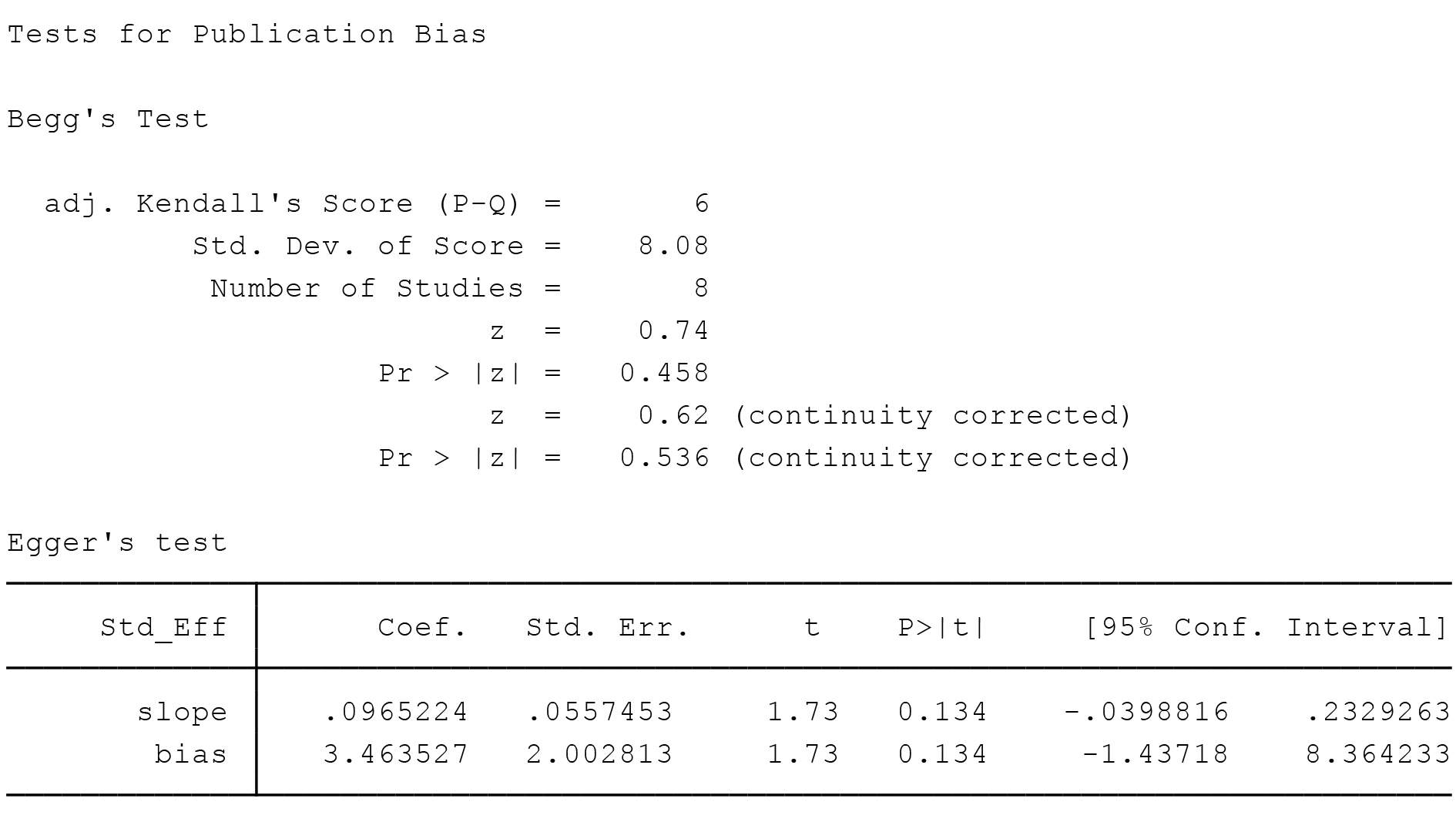

Supplement: Supplementary file 4 [file Image_3.TIF]
